# The Carotid Siphon as a Pulsatility Modulator for Brain Protection: Role of Arterial Calcification Formation

**DOI:** 10.3390/jpm15080356

**Published:** 2025-08-04

**Authors:** Pim A. de Jong, Daniel Bos, Huiberdina L. Koek, Pieter T. Deckers, Netanja I. Harlianto, Ynte M. Ruigrok, Wilko Spiering, Jaco Zwanenburg, Willem P.Th.M. Mali

**Affiliations:** 1 Department of Radiology, University Medical Center Utrecht, Utrecht University, 3508 GA Utrecht, The Netherlands; 2 Department of Epidemiology, Erasmus Medical Center, 3015 GD Rotterdam, The Netherlands; 3 Department of Radiology and Nuclear Medicine, Erasmus Medical Center, 3015 GD Rotterdam, The Netherlands; 4 Department of Epidemiology, Harvard T.H. Chan School of Public Health, Boston, MA 02138, USA; 5 Department of Geriatric Medicine, University Medical Center Utrecht, Utrecht University, 3508 GA Utrecht, The Netherlands; 6 Department of Neurology, University Medical Center Utrecht, Utrecht University, 3508 GA Utrecht, The Netherlands; 7 Department of Vascular Medicine, University Medical Center Utrecht, Utrecht University, 3508 GA Utrecht, The Netherlands; 8 Center for Image Sciences, University Medical Center Utrecht, Utrecht University, 3508 GA Utrecht, The Netherlands

**Keywords:** carotid artery, arterial calcification, physiology, stroke, dementia

## Abstract

A healthy vasculature with well-regulated perfusion and pulsatility is essential for the brain. One vascular structure that has received little attention is the carotid siphon. The proximal portion of the siphon is stiff due to the narrow location in the skull base, whilst the distal portion is highly flexible. This flexible part in combination with the specific curves lead to lower pulsatility at the cost of energy deposition in the arterial wall. This deposited energy contributes to damage and calcification. Severe siphon calcification stiffens the distal part of the siphon, leading to less damping of the pulsatility. Increased blood flow pulsatility is a possible cause of stroke and cognitive disorders. In this review, based on comprehensive multimodality imaging, we first describe the anatomy and physiology of the carotid siphon. Subsequently, we review the in vivo imaging data, which indeed suggest that the siphon attenuates pulsatility. Finally, the data as available in the literature are shown to provide convincing evidence that severe siphon calcifications and the calcification pattern are linked to incident stroke and dementia. Interventional studies are required to test whether this association is causal and how an assessment of pulsatility and the siphon calcification pattern can improve personalized medicine, working to prevent and treat brain disease.

## 1. Introduction

**Figure 1:**
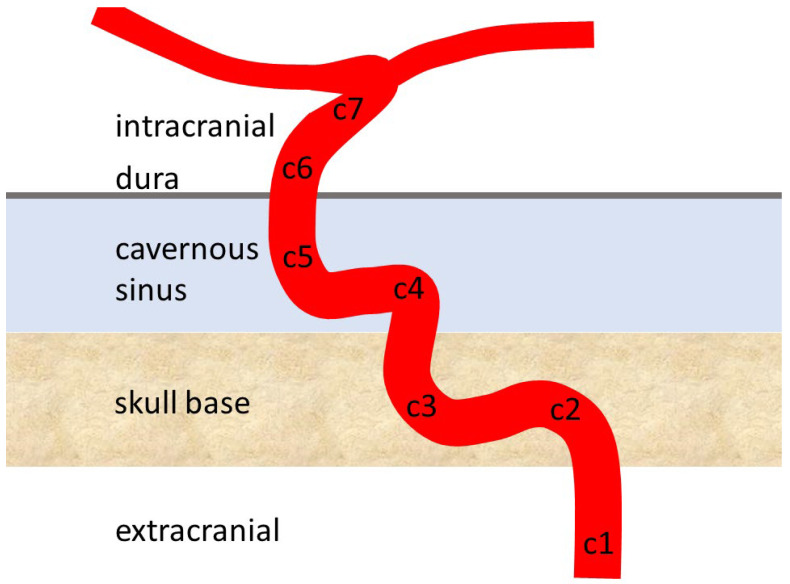
Structure of the carotid siphon. This illustrates the general shape of the siphon, although there is personal variation and even individual shapes, such as C, V, and S-shapes, which have been recognized. Please note that C2 and C3 are in the skull base and cannot distend, while C4 and C5 are within venous blood without attachment and thus more flexible. In addition to the form (bends), the C4 and C5 parts may dampen pulsatility.

The human carotid siphon is a highly curved part of the internal carotid artery, subdivided into seven segments (Figure 1). C1 is extracranial, while C2 and C3 are located in the skull base, fixed in the bone and unable to pulsate (Figure 1) [1,2]. C4 and C5 are intracranial within the cavernous sinus, allowing free movement and pulsation due to a lack of attachments. After crossing the dura mater, C6 and C7 lie intradurally before bifurcating into the anterior and middle cerebral arteries. The siphon wall is thin, lacking an external elastic lamina [3,4], and is densely innervated, partly by parasympathetic nerve fibers, but their function is unknown [5,6,7,8].

Several mechanisms exist to protect the brain against pulsatile flow, including aortic elasticity and pressure dissipation by the circle of Willis [9,10]. Hunter already postulated in 1793 that there is a specific role for the carotid siphon [11]. Arterial curves generally reduce pulsatility by converting the blood’s inertia into vessel wall strain through centrifugal forces, stretching the vessel longitudinally rather than radially (Windkessel effect) [12,13,14]. Both mechanisms reduce downstream pulsatility and the Pulsatility Index (PI), which can be measured via flow, area, or velocity. The PI is a reflection of flow pulsatility. Velocity PI (vPI) and area distensibility index (aPI) indicate local arterial stiffness and can be measured using 2D or 3D phase-contrast MRI (PCMRI) [15]. Schubert et al. [16] used 3D-PCMRI at 3 Tesla in 17 healthy volunteers and found PI decreases between levels C4 and C7 of the internal carotid artery (ICA), suggesting that curves in the cavernous sinus reduce pulsatility. A recent study [17] using 2D-flow PCMRI in 118 healthy individuals showed that vPI increased from C1 to C3, with no net change between C1 and C7, suggesting that the rigid carotid canal at C3 restricts wall motion and increases the vPI, while the siphon reabsorbs kinetic energy, reducing the vPI again. Thus, the canal and siphon function as a unit to redistribute energy, locally damping flow pulsatility.

Pulsatility attenuation in the carotid siphon declines with age and calcifications. In a study of 17 cerebral small vessel disease (cSVD) patients and 17 controls using 4D-PCMRI at 7T, both groups showed an increased vPI and a decreased aPI in the C3–C4 segment. However, the vPI attenuated from C1 to C7 in controls but not in cSVD patients; instead, the vPI increased from C1 to C7 in cSVD, indicating abnormal pulsatility regulation [18,19]. Another study of 50 pseudoxanthoma elasticum (PXE) patients and 40 controls using 2D-flow MRI at 3T found a higher vPI and reduced distensibility in PXE patients, but attenuation between C4 and the MCA was similar in both groups. Subsequent analysis showed that PXE patients had a higher flow PI, associated with severe siphon calcification, a reduced gray matter volume, more white matter lesions, and lacunar infarctions [19,20,21,22,23,24].

Overall, the healthy carotid siphon dampens kinetic energy pulsatility, but aging and calcifying diseases cause siphon stiffening, preventing this attenuation and transmitting harmful pulsatility to the cerebral tissue, contributing to cSVD-related damage [9,10,11].

Remarkably, internal elastic lamina calcification in the siphon can occur in children [25], reflecting the repair of damaged elastic fibers from energy deposition [7,26,27]. Early studies showed two types of siphon calcification: atherosclerotic intimal calcifications and elastic lamina or medial calcifications (diffuse, ring-like) [28,29]. On imaging, atherosclerotic calcifications were scattered and focal, while medial calcifications were diffuse and tended to form rings [30]. The degree of calcification is often graded as none, mild, moderate, or severe [28,29].

**Figure 2:**
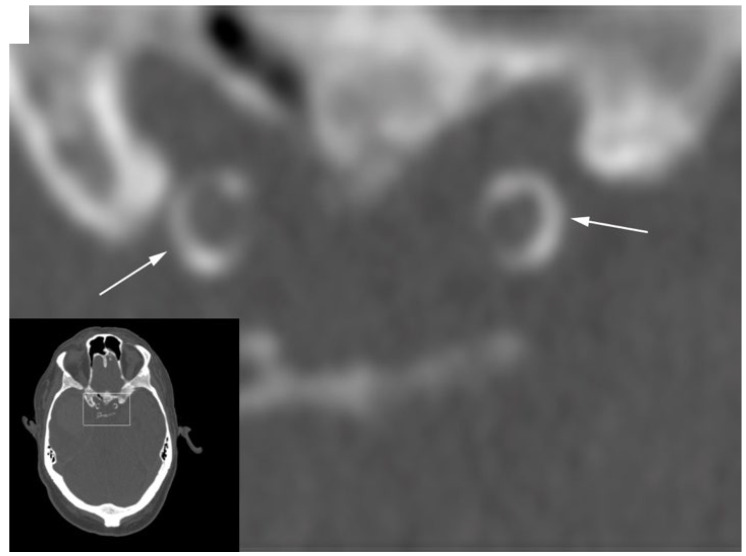
Bilateral circular calcifications of the carotid siphon.

Woodcock et al. [31] proposed a qualitative CT grading scale based on continuity and thickness, later modified by Babiarz et al. [32] to include the circumferential extent (<90°, 90–270°, 270–360°) and thickness (0 to >3 mm). The Rotterdam Study introduced semi-automatic quantification, defining calcifications as pixels >130 HU (Figure 2) [33]. Calcification was defined as pixels above 130 HU localized in the arterial wall, as proposed for the coronary arteries by Agatston [34].

Recently, a CT-based scoring system has differentiated intimal from medial calcifications, as validated against histology [26]. In one study, 72% of siphon calcifications were medial (elastic lamina) and only 28% atherosclerotic intimal, suggesting that siphon calcifications in Caucasians are often non-atherosclerotic. The scoring system assesses circularity, thickness, and morphology, with ≥7 points indicating medial calcification dominance [35]. While it cannot differentiate limited calcifications, it reliably categorizes dominant patterns in cohorts.

Although, recently, the carotid siphon and its calcification have gotten more attention, and our knowledge of the hemodynamic meaning of the siphon and the effects of calcification have increased, little is known about the determinants of carotid siphon calcification and its association with clinical disease. This literature review aims to summarize the most up-to-date literature on the carotid siphon and to place the findings of risk factors and calcifications in perspective.

## 2. Literature Search Methods

The articles included in this narrative review were selected by means of author expertise, which was supplemented by searches in PubMed up to September 2024. Articles describing the carotid siphon and physiology, calcifications, or risk factors were included by the first and senior authors (P.A.d.J. and W.P.Th.M.). Case reports were excluded. A search was performed using a combination of the terms (“carotid siphon” OR “intracranial carotid”) AND (“pulsatility” OR “distensibility” OR “elasticity” OR “calcification” OR “risk factor”). In addition, we performed a citation review of the reference lists of included primary and review articles. Included articles were published in English, and no other languages were considered.

## 3. Determinants of Carotid Siphon Calcification

Studies have investigated determinants of siphon calcification presence, volume, and severity. Data on intimal versus medial calcification dominance are growing, though older studies remain hard to interpret due to differing risk factors [36,37,38].

Age is the most investigated determinant. Bos et al. [39] found calcification in up to 80% of a population (mean age 69.6 years). In a CT study of 1868 trauma patients, mild calcifications appeared in up to 10% before age 30, increasing to 30% by the fifth decade, then declining in the tenth. Moderate calcifications emerged in the fifth decade (12%), peaking at 55% in the ninth. Severe calcifications began in the sixth decade (3%), rising to 47% by the tenth decade [25].

The Rotterdam cohort applied CT scoring in 2391 stroke-free participants (mean age 69.6 years): intimal calcifications were present in 37% under age 65, decreasing to 13% over 85; medial calcifications increased from 27% under 65 to 79% over 85, with 98% having any calcification by age 85+ [38].

Beyond age, determinants include diabetes, renal dysfunction, hypertension, and aortic stiffness [40,41,42,43,44,45,46] (Table 1). Sex differences are minimal [47], the influence of race is largely unknown, and associations with obesity, smoking, and dyslipidemia are inconsistent [39,42,43,48,49,50]. More or less consistent positive associations are found with diabetes mellitus, renal dysfunction, hypertension, and aortic stiffening [49,50,51,52,53,54,55,56]. The heritability of siphon calcification was in one study estimated to be large [57], while less conventional risk factors, including aldosterone, 3-hydroxybutyrade, homocysteine, and missing teeth, remain underexplored [58,59,60,61,62,63].

## 4. Clinical Disease Associations of Carotid Siphon Calcification

A large number of studies have investigated siphon calcifications in relation to intracranial disease outcomes [64,65,66]. Most studies used the visual or quantitative calcification severity, but some determined whether there was atherosclerotic versus internal elastic lamina calcification (Table 2).

### 4.1. Subarachnoid Hemorrhage

The few available studies suggest that the siphon calcification burden is associated with poor outcomes in subarachnoid hemorrhage. Engel et al. observed in 716 patients that severe siphon calcifications were associated with poor outcomes, but less vasospasm [98]. The authors suggested that stiff arteries may prevent vasospasm, but apparently not a poor outcome [67]. One case–control study showed that siphon calcifications were not associated with a ruptured aneurysm status [68].

### 4.2. Prevalent and Incident Stroke

Many studies have demonstrated that siphon calcifications are independently associated with the stroke presence and incident strokes [38,69,70,72,73,74,76,77,79,81,84,86,88,90,92,93,94,95,97]. In 1975, Scotti et al. investigated over 5000 skull radiographs. They found siphon calcifications to be associated with stroke, and intimal and medial patterns had a similar effect size, but separation was suboptimal on the radiographs [97]. The early CT studies were often in small samples [95]. A study in 175 acute ischemic stroke patients and 182 controls found an odds ratio (OR) of 3.17 (95%CI: 1.25–8.04) for siphon calcification [93]. In 156 consecutive TIA patients, the calcium score was significantly higher in those with recurrence after multivariable adjustment (aOR 1.25, 1.01–1.55) [81]. In 2014, Bos et al. [88] demonstrated in 2323 stroke-free persons that larger siphon calcification volumes were related to a higher risk of incident stroke. The adjusted hazard ratio (aHR) per SD increase in calcification volume was 1.43 (95%CI: 1.04–1.96). Later, the calcifications were visually scored and associated with incident stroke (aHR for intimal: 2.11, lamina: 2.66, and mixed subtype: 2.57) [38]. Some data suggest sex-specific effects. In a study with 1184 women and 983 men, siphon calcification was associated with a higher risk of stroke (aHR 1.40, 95%CI: 1.03–1.90) in women, but not in men [76]. The overall conclusion is that there is substantial evidence that siphon calcification is independently associated with stroke [69,70,73,79,84,90,92,94], especially for lamina calcifications [72,74,77,86].

### 4.3. White Matter Disease, Cortical Atrophy, Cognition, and Dementia

Several studies have investigated the association between siphon calcification and damage to brain tissue and/or cognitive function [36,37,49,75,80,86,87,91,92,96,99]. Siphon calcification is associated with cerebral atrophy, white matter lesions, impaired cognitive function, and incident dementia [99].

In 1977, in 173 patients with Parkinson’s disease, cerebral atrophy was shown to be associated with siphon medial calcification [96]. In 2008, in 65 consecutive patients, severe siphon calcification was associated with central atrophy [92]. In 159 ischemic stroke patients, Chung et al. [91] observed that siphon calcification was associated with periventricular white matter lesions (aOR 2.62, 95%CI: 1.24–5.53) and deep white matter lesions (aOR 3.25, 95%CI: 1.53–6.89). In 1458 stroke-free participants of the Rotterdam Study, it was shown that internal elastic lamina calcification, considered a proxy for arterial stiffness, was the leading mechanism explaining the link between blood pressure and cSVD [36].

Regarding cognition and clinical disease, in a large population study, Van den Beukel et al. showed that non-atherosclerotic siphon calcification increased the risk of incident dementia (the hazard more than doubled in the upper tertile), while atherosclerotic calcifications did not increase the risk of dementia [37]. Mediation analysis indicated that the risk of non-atherosclerotic calcification for dementia was mediated via white matter lesions. In 1992, this could not be confirmed among memory clinic patients, as siphon calcification was not associated with dementia [49], but the high prevalence of calcifications (95%) and selection bias (71% had mild cognitive impairment or dementia) may have played a role. Many other studies on siphon calcification severity support the association with a loss of cognition and dementia independent of beta amyloid [75,80,86,87,99].

### 4.4. Siphon Calcification and Clinical Interventions

In the MR CLEAN trial, including 500 stroke patients, post hoc analyses were performed that specifically focused on siphon calcification. It was found that stroke patients with a predominant medial calcification subtype benefited more from endovascular treatment that than those with intimal calcification [82]. An important explanation for this, at first sight, paradoxical finding may be found in the presence or absence of good cerebral collaterals. Indeed, the same investigators showed in 2701 stroke patients that patients with a predominant medial calcification subtype showed a collateral status that was comparable to patients without calcification [77], and that patients with intimal calcification were more likely to have good collaterals, which has been confirmed by some (e.g., [83]), but not all studies (e.g., [78]). Studies on whether siphon calcification is associated with recanalization success are inconclusive [71,85]. Overall, there is some evidence that the effect of stroke therapy is better in medial siphon calcification.

## 5. Therapeutic Options for Siphon Calcification

Under the assumption that siphon calcification plays a causal role in the development of brain diseases, it could be useful to slow calcification development. Theoretically, treatment and prevention of hypertension, diabetes mellitus, and renal dysfunction may help, but trials are disappointing [100,101,102,103]. Another option is targeting the calcification itself, as already performed by Kramsch et al.’s study in monkeys, reported in *Science* in 1981 [104]. A recent meta-analysis showed that first-generation bisphosphonates had a beneficial effect on vascular calcification [105]. The most promising bisphosphonate is etidronate, for which two small RCTs are available [106,107]. The first study concerns 74 PXE patients, who were randomized to etidronate or a placebo. Etidronate slowed arterial calcification progression, which was further supported during post-trial access [108]. The second RCT [109] concerned 109 patients with hypercholesterolemia randomized for atorvastatin, etidronate, or both. The endpoint was the percentage change in maximal vessel wall thickness in the thoracic and abdominal aorta. In a post hoc analysis, it was shown that etidronate primarily reduced calcified abdominal aortic plaques.

## 6. Conclusions

**Figure 3:**
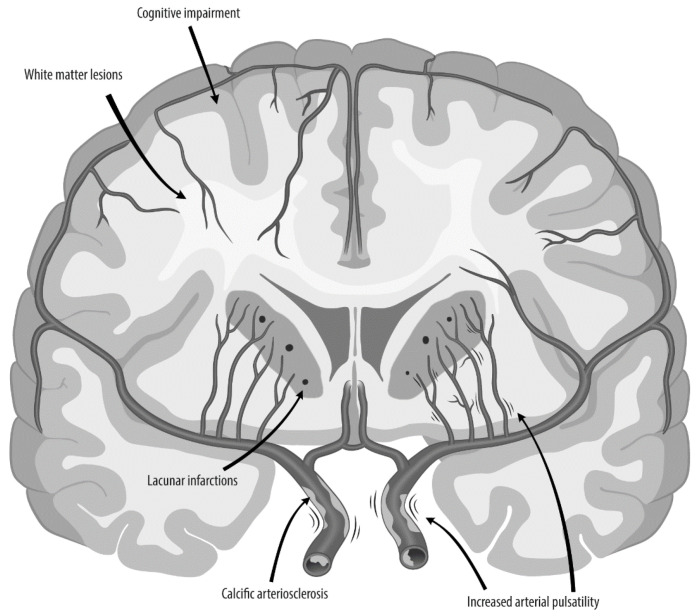
From siphon arteriosclerosis to symptomatic disease. A schematic overview of the literature on siphon calcification and a hypothesized temporal link. Siphon calcifications are associated with increased arterial pulsatility, lacunar infarctions, white matter disease and loss of cognition, incident dementia, and stroke. This suggests that targeting calcific arteriosclerosis of the siphon could aid personalized medicine of brain diseases.

Our proposal is that the bends of the siphon and the flexible distal part function to protect the brain, as suggested by Hunter in 1793 [11]. In the siphon, the most common structural abnormality is elastic lamina calcification, which is linked to an altered physiology and multiple adverse outcomes, such as stroke and dementia. Severe calcification of the distal carotid siphon may thus be an often-overlooked causal step in the process leading to cerebrovascular disease (Figure 3), in addition to upstream (aortic stiffening) [9] and downstream factors [110], such as pressure dissipation by the circle of Willis [10]. As it is possible that siphon calcification plays a causal role in the vicious circle of vascular stiffening, stroke and dementia research requires intervention studies that slow, prevent, or reverse siphon calcification.

## Figures and Tables

**Table 1:** Determinants of carotid siphon calcification.

Author	Population	Association with Calcification Presence, Calcification Severity, or Calcification Pattern
Lucci C, 2024 [41]	475 cardiovascular patients	Intimal calcification: older age, systolic blood pressure (women), glucose levels (women), current smoking (men), eGFR ≤60 mL/min/1.73 m^2^ (men). Medial calcification: older age, vitamin K antagonists (women), lipid-lowering drugs (women)
Gozdalski J, 2024 [42]	65 patients with ischemic stroke	Age, diabetes, smoking, carotid-femoral PWV (higher in medial compared to intimal pattern)
Oge DD, 2024 [43]	250 stroke patients	Lower bone density (T-score) only with intimal calcification pattern. Intimal: age, male, diabetes, albumin. Medial: age, male, diabetes, coronary artery disease, albumin
Singh SS, 2023 [44]	2354-member general population sample	Lipoprotein A
Del Brutto OH, 2020 [45]	581 Amerindian community members aged ≥60 years	Increasing age, high fasting blood glucose, >10 enlarged basal ganglia-perivascular spaces and non-lacunar strokes
Voigt S, 2021 [47]	1397 stroke patients	Male gender only for intimal pattern
Golüke NMS, 2022 [49]	1992 memory clinic patients	Age, male (intima), diabetes mellitus (medial), hypertension (intimal), smoking (intimal), stroke
Peeters MTJ, 2019 [40]	376 patients with nontraumatic intracerebral hemorrhage	Age
Sedaghat S, 2019 [53]	2241-member general population	Lower eGFR and a higher albumin-to-creatinine ratio
de Onofre NML, 2021 [58]	284 dental clinic patients aged >40 years	More missing teeth, older age, hypertension, diabetes, coagulation disorders, antihypertensive drugs, C4 segment
AlSakr A, 2021 [59]	208 dental clinic patients aged <30 years	Periodontitis, hypertension, hyperlipidemia, increasing age, male gender, cardiovascular history
Kockelkoren R, 2018 [25]	1868 trauma patients	Age
Zhang S, 2019 [1]	207 acute ischemic stroke patients	Aldosterone
Vos A, 2018 [50]	1132 stroke patients	Age, pulse pressure, and family history (any calcification). Intimal: smoking, hypertension. Medial: diabetes, previous vascular disease
Del Brutto OH, 2018 [46]	437 Amerindian community members aged ≥60 years	Carotid-femoral PWV
Vojinovic D, 2018 [61]	1111-member general population	3-hydroxybutyrate
Olatunji RB, 2018 [51]	130 adults with acute ischemic stroke	Age, hypertension, diabetes mellitus, hyperlipidemia, alcohol use
Del Brutto OH, 2017 [52]	663 adults from general population	Brachial pulse pressure
Kim JM, 2016 [62]	1193 patients with infarction or TIA	Serum homocysteine
Adams HH, 2016 [57]	2034-member general population	47% heritability, locus 9p21.3 (rs1537372), 11p11.2 (rs11038042)
Yilmaz A, 2015 [54]	319 ischemic stroke patients	Age, diabetes, coronary artery disease (univariate hypertension and inverse relation with smoking)
Bos D, 2012 [39]	2495-member general population	Age, cardiovascular history, excessive alcohol intake (men), smoking (men), diabetes (women), hypertension (women), less obesity (women)
Iwasa Y, 2012 [55]	107 hemodialysis patients, 43 controls	Hemodialysis
Mak HK, 2009 [56]	60 Chinese patients with TIA or minor stroke	Age, diabetes
de Weert TT, 2009 [48]	406 patients with ischemic cerebrovascular disease	Age, male, smoking, hypercholesterolemia, history of cardiovascular disease
Chen XY, 2006 [63]	490 patients referred for brain CT	Age, history of ischemic stroke, white blood cell count

**Table 2:** Outcomes associated with carotid siphon calcification.

Author	Population	Association with Calcification Presence, Calcification Severity, or Calcification Pattern
Engel 2024 [67]	716 patients with subarachnoid hemorrhage	Calcification score was related to adverse outcome (aOR 4.06) and early ischemia (aOR 1.58). Calcification score was protective for vasospasm.
van der Toorn JE, 2020 [64]	1239 females and 1118 males, general population	All-cause mortality in males (aHR 1.34) and cardiovascular mortality in males (aHR 2.11) and females (aHR 1.95).
Kamphuis MJ, 2024 [68]	150 with unruptured and 150 with ruptured aneurysms	Siphon calcifications are not associated with rupture status.
Zhu J, 2023 [69]	207 patients with anterior circulation stroke	Medial siphon calcification pattern is associated with poor stroke outcome (sOR: 7.418).
Van den Beukel T, 2024 [37]	2339-member general population, stroke- and dementia-free	Siphon calcification presence increased risk of incident dementia (aHR 1.53).
Shimoyama T, 2023 [70]	375 acute ischemic stroke patients	Calcification volumes in the siphon associated with large artery stroke in younger patients (aOR; 2.89).
Xia J, 2023 [71]	177 patients with non-acute occlusion	Siphon calcification was associated with successful recanalization.
Mazzacane F, 2023 [65]	485 ischemic stroke patients	Intimal calcification was associated with lacunar stroke etiology (aOR 2).
Del Brutto OH, 2022 [72]	778 persons who underwent head CT	Visual calcification score of moderate or severe was associated with all-cause mortality (aHR 1.82).
Hou D, 2022 [73]	310 stroke patients	Siphon calcification presence and lower density was independently associated with stroke progression (aOR density 1.23).
Shen Y, 2022 [74]	156 stroke patients who received IVT	Modified Woodcock score associated with poor stroke outcome (aOR = 1.35) and death (aOR = 2.41).
Rahmani F, 2022 [75]	Case–control of 230 subjects who underwent PET/CT	Agatston score of the siphon was not associated with cognitive decline.
Van den Beukel TC, 2022 [38]	Population-based cohort of 2391 stroke-free participants	All siphon calcification subtypes were associated with a higher risk of stroke (aHR intimal: 2.11, elastic lamina: 2.66, mixed 2.57).
Van der Toorn JE, 2021 [76]	1184 women and 983 men, population cohort	Especially in women, siphon calcification score was associated with myocardial infarction (MI), other coronary heart disease mortality, and stroke (aHR women 1.62, men 1.26).
Luijten SPR, 2021 [77]	2701 stroke patients who underwent EVT	Patients with intimal siphon calcification pattern benefit more from an extensive collateral circulation in terms of outcome.
Kouw F, 2021 [78]	982 stroke patients	Intravenous thrombolysis was significantly associated with favorable clinical outcome in medial siphon calcifications.
Pektezel MY [79]	201 patients with intracerebral hematoma	No clear association with hematoma expansion, mortality, or adverse outcome.
Golüke N, 2021 [49]	1992 memory clinic patients	No independent association between siphon calcification (pattern or severity) and cognitive function.
He XW, 2019 [66]	32 acute ischemic stroke patients	Siphon calcification severity was not independently associated with stroke severity, hemorrhagic transformation, functional outcome, or mortality.
Cho N, 2019 [80]	69 patients with chronic kidney disease	Siphon calcium score was not significantly associated with cognitive impairment (aOR 2.65 (0.49–16.04), but power was limited.
Kong WY, 2019 [81]	156 consecutive TIA patients	A higher CT calcium score was significantly associated with recurrent ischemic events (aOR 1.25).
Compagne K, 2018 [82]	500 stroke patients included in the MR CLEAN study	Siphon medial calcification patients had better functional outcome with endovascular therapy (aOR 2.32) compared to those with intimal calcifications (aOR 0.82).
Gocmen R, 2018 [83]	91 consecutive acute anterior circulation stroke patients	Medial dominance of siphon calcification tended to be associated with less early response to intravenous thrombolysis (*p* = 0.052), but not to later outcome or hemorrhagic transformation.
Tábuas-Pereira M, 2018 [84]	396 consecutive ischemic stroke patients	Siphon calcification score was only associated with mortality (aOR 1.10), not with other adverse outcomes.
Hernández-Pérez M, 2017 [85]	194 patients admitted to a stroke unit	Siphon calcification associated with incomplete revascularization (aOR 0.73) and with poor outcome (aOR 1.31).
Del Brutto, 2016 [86]	584 persons without previous stroke	Inverse relation between siphon calcification severity and cognition (adjusted Beta −2.04, −3.76 to −0.33).
Kao HW, 2015 [87]	579 patients scanned for multiple indications	Lower cognitive function association with 100-point increment of siphon Agatston score (aOR 1.06).
Bos D, 2014 [88]	2323 stroke-free persons	Siphon calcification volume was related to incident stroke (aHR per SD 1.43).
Lin TC, 2013 [89]	297 stroke patients	Moderate to severe siphon calcification risk factor for hemorrhagic transformation (aOR 2.52).
Hong NR, 2011 [90]	445 patients who underwent CT and MRI	Carotid siphon calcification severity with lacunar infarcts (aOR 1.29, 1.15–1.45).
Chung PW, 2010 [91]	159 acute ischemic stroke patients	Siphon calcification was associated with periventricular white matter lesions (aOR 2.62), deep white matter lesions (aOR 3.25), and lacunar infarcts (aOR 3.09).
Erbay S, 2008 [92]	65 acute patients who underwent CT and MRI	Siphon calcification was associated with central atrophy (after adjustment for age), but not with cortical atrophy.
Chen XY, 2007 [93]	175 stroke cases and 182 controls	Association between siphon calcification and ischemic stroke (aOR = 3.172).
Erbay S, 2007 [94]	65 acute patients who underwent CT and MRI	Severe siphon calcification was associated with acute small vessel infarcts after multivariable adjustment (*p* = 0.002).
Taoka T, 2006 [95]	72 patients with unenhanced CT and angiography	N = 7 experienced a stroke. Mean calcium score of the no-stroke group was 88 (SD 171) and of the stroke group was 312 (SD 539).
Schneider E, 1977 [96]	173 treated and untreated Parkinsonian patients	Medial calcification is associated with brain atrophy, but not with Parkinsonism.
Scotti G, 1975 [97]	5570 patients who underwent skull radiography	Both intimal and medial calcifications were associated with stroke occurrence, with a similar effect size.

## Data Availability

Not applicable.
